# Comparison of the efficacy and safety of moclobemide and imipramine in the treatment of depression in Indian patients

**DOI:** 10.4103/0019-5545.55951

**Published:** 2005

**Authors:** Ajit Avasthi, Parmanand Kulhara, Gagandeep Singh, Rajni Sharma, Rajinder P. Kaur

**Affiliations:** * Additional Professor of Psychiatry, Postgraduate Institute of Medical Education and Research; ** Professor and Head of Psychiatry, Postgraduate Institute of Medical Education and Research; ***** Clinical Psychologist, Department of Psychiatry Postgraduate Institute of Medical Education and Research; **** Play Therapist, Department of Paediatrics Postgraduate Institute of Medical Education and Research; *** Senior Lecturer of Psychiatry, Government Medical College and Hospital, Chandigarh

**Keywords:** Moclobemide, depression, monoamine oxidase-A inhibitor

## Abstract

**Background:**

Moclobemide, a benzamide, is one of the new-generation monoamine oxidase-A inhibitors (MAO-AIs) which belongs to the class of reversible inhibitors of monoamine oxidase (RIMA). Numerous studies have shown that moclobemide is well-tolerated and as effective as other antidepressants. So far, there is no evidence available regarding its efficacy and tolerability in India.

**Aim:**

To compare the efficacy and safety of moclobemide with that of imipramine, a tricyclic antidepressant, in the treatment of major depressive disorder.

**Methods:**

In this prospective study, patients (*n*=60) were randomly assigned to treatment with moclobemide or imipramine for 6 weeks. Efficacy of the drugs was assessed by observing the mean change in scores on the Hamilton Depression Rating Scale (HDRS) and the Montgomery–Asberg Depression Scale (MADRS).

**Results:**

Both the groups showed significant decrease in scores at the end of 6 weeks. Patients who received moclobemide had a better side-effect profile.

**Conclusion:**

Moclobemide is an effective antidepressant and is better tolerated than imipramine.

## INTRODUCTION

Depression is a major public health problem, causing subjective distress, impaired functional capacity, secondary mental and somatic complications and increase in mortality. An accurate diagnosis followed by efficient treatment can improve the outcome.^1^

Classically, tricyclic antidepressants (TCAs) and monoamine oxidase inhibitors (MAOIs) have been used to treat depression. For the past two decades, researchers have aspired to develop clinically effective antidepressants with a more rapid onset of action and/or less troublesome side-effects. These efforts led to the development of selective serotonin reuptake inhibitors (SSRIs), selective norepi-nephrine reuptake inhibitors (SNRIs) and reversible inhibitors of monoamine oxidase (RIMA).^2^

Although MAOIs were among the first substances to be used as antidepressants, due to their ‘cheese effects’, they largely fell into disuse in the 1960s.^3^ Moclobemide, a benzamide, is one of the new generation MAOIs which belongs to the class of RIMA. It selectively inhibits monoamine oxidase-A (MAO-A) and does not affect other enzyme systems.^2^ The inhibition of monoamine oxidase-A, which is reversible, imparts two important clinical characteristics to this drug. First, minimal potentiation occurs in case of high availability of tyramine (as a substrate in food), hence, the risk of hypertensive crisis after intake of tyramine-rich food is negligible.^4^ Second, after termination of moclobemide treatment, MAO activity returns to normal within one day.^5^ Both animal and human pharmacological studies have established that no clinically significant interaction occurs if moclobemide is taken after consumption of tyramine in physiological amounts.^4^,^6^,^7^ In addition, its non-affinity for muscarinic, dopaminergic, serotoninergic, opioid or benzodiazepine receptors protects against the development of a host of adverse reactions seen in case of TCAs.^2^

Previous studies comparing moclobemide and placebo in patients with major depressive disorder found moclobemide to be significantly better than the placebo.^8^–^10^ Moclobemide is found to be well-tolerated and equally effective as other antidepressants, i.e. heterocyclic compounds such as imipramine,^11^,^12^ amitriptyline,^13^,^14^ clomipramine,^15^,^16^ SSRIs such as fluoxetine,^17^,^18^ sertraline^19^ and older MAO inhibitors such as tranylcypramine.^20^ These findings were reconfirmed in a meta-analysis of RIMA-type A moclobemide and brofarmine in the treatment of major depression.^21^ Gagiano *et al*.^22^ established the usefulness and safety of moclobemide for continuation treatment of major depressive episodes. The most commonly reported adverse effects of moclobemide are insomnia (13%), nausea (11%), headache (11%), dizziness (6%), agitation (3%) and diarrhoea (3%).^23^

Despite overwhelming data from western countries, there is no evidence available from India regarding its efficacy and tolerability. We evaluated the efficacy and safety of moclobemide in comparison with the TCA imipramine in the treatment of depression in Indian patients.

## METHODS

Patients attending the outpatient clinic of a tertiary-care teaching hospital were selected for the study. Men and women between the ages of 18 and 50 years, fulfilling the ICD-10 criteria for major depression,^24^ and having a minimum score of 18 on the 24-item Hamilton Depression Rating Scale (HDRS)^25^ and 25 on the Montgomery–Asberg Depression Rating Scale (MADRS)^26^ were included in the study. Patients who had been administered a clinically effective dose of antidepressants in the preceding 2 weeks, electroconvulsive therapy (ECT) in the preceding 3 months, those on MAO inhibitors, and those with concurrent physical or co-morbid psychiatric illness (including substance abuse) were excluded from the study. All patients gave their informed consent to participate in the study. The approval of Ethics Committee and permission from the Drug Controller General of India (DCGI) were obtained before initiating the study.

It was an open, randomized, comparative study of 6 weeks' duration. Of the 60 patients enrolled in the trial, 30 were randomized to receive moclobemide and 30 imipramine. The sociodemographic data of both the groups revealed that the majority of patients were men (55%), married (80%), educated up to matriculation (71%), employed (55%) and hailed from nuclear families (55%) of urban background (55%). The two groups did not differ significantly in any of these sociodemo-graphic variables.

The patients underwent a detailed physical examination, and clinical as well as laboratory investigations. Moclobemide was started at a dose of 300 mg daily (2 × 150 mg) or 75 mg imipramine daily as per the study protocol. The dosage was increased at an interval of not less than 1 week to a maximum of 600 mg for moclobemide daily or 300 mg for imipramine daily if there was no clinical improvement/clinical deterioration, or less than 20% improvement in the scores of the HDRS during the study period.

### Study population

Twenty patients (33.3%) did not complete the 6-week treatment period; 9 (30%) on moclobemide, 11 (36.7%) on imipramine. In moclobemide group, of the 9 patients, 3 were lost to follow-up, while 2 each had poor compliance, refused treatment and developed a new set of symptoms. In the imipramine group, 2 patients discontinued treatment due to adverse effects, 3 were lost to follow-up and 3 each refused treatment and developed a new set of symptoms.

Of the 30 patients in each group, 24 in each group fulfilled the criteria for ‘Intent to treat’ patients. ‘Intent to treat’ was defined as patients either completing the study protocol or had received the minimal possible intended dose and did not include those patients who had poor compliance/refused treatment or developed a new set of symptoms.

The safety and tolerability of treatment were evaluated at the end of weeks 1, 2, 4, and 6 based on vital signs (heart rate, blood pressure), laboratory values (baseline and post-treatment) and adverse effects.

The efficacy was evaluated at the end of week 2, 4, and 6 based on scores on the HDRS and MADRS. A responder was defined as one showing a decrease of at least 50% in the scores on the MADRS or the HDRS from the baseline.

## RESULTS

### Efficacy

#### 1. Hamilton Depression Rating Scale (HDRS)

In the ‘treatment completers’, there was a steady decrease in depression rating in both the treatment groups, with the mean total score falling from 29.14 (SD±5.64) at baseline to 12.95 (SD±8.17) at the end of 6 weeks in the moclobemide group, and from 29.68 (SD±4.43) to 8.52 (SD±7.16) in the imipramine group. The change in both the groups from the baseline to the end of week 6 was statistically significant (p<0.001). The mean changes and percentage reductions of the HDRS scores in week 0–2, week 2–4, week 4–6 and week 0–6 were significant (p<0.001) but comparable across both the groups (Table 1).

**Table 1:** Mean changes and percentage reduction in the scores on the Hamilton Depression Rating Scale (HDRS) in ‘treatment completers’

	Moclobemide (*n*=21)		Imipramine (*n*=19)	
		
Duration (in weeks)	Mean changes	Reduction (%)	Mean changes	Reduction (%)
Baseline–week 2	7.48	25.66*	11.47	38.64*
Week 2–week 4	5.62	25.94*	5.16	28.33*
Week 4–week 6	3.09	19.26*	4.53	34.71*
Baseline–week 6	16.19	55.56*	21.16	71.29*

* p<0.001

In the ‘Intent to treat’ patients, the mean scores on the HDRS fell from 29.50 (SD±5.05) at baseline to 14.04 (SD±8.96) at the end of 6 weeks in the moclobemide group and from 30.25 (SD±4.28) at baseline to 11.71 (SD±8.43) at the end of 6 weeks in the impramine group. The change in both the groups from baseline to the end of week 6 was statistically significant (p<0.001). The mean changes and percentage reductions in the HDRS scores in week 0–2, week 2–4, week 4–6 and week 0–6 were significant (p<0.001) but comparable across both the groups (Table 2).

**Table 2:** Mean changes and percentage reduction in the scores on the Hamilton Depression Rating Scale (HDRS) in the ‘Intent to treat patients’ group

	Moclobemide (*n*=24)		Imipramine (*n*=24)	
		
Duration (in weeks)	Mean changes	Reduction (%)	Mean changes	Reduction (%)
Baseline–week 2	7.42	25.14*	11.67	38.57*
Week 2–week 4	5.08	23.00*	4.04	21.74*
Week 4–week 6	2.96	17.41*	2.83	19.46*
Baseline–week 6	15.46	52.41*	18.54	61.29*

* p<0.001

#### 2. Montgomery–Asberg Scale (MADRS)

In ‘treatment completers’ the mean total score fell from 36.47 (SD±6.58) at baseline to 18.42 (SD±11.47) at week 6 in the moclobemide group, and from 36.26 (SD±5.50) to 12.15 (SD±8.57) in the imipramine group. Although the changes between baseline and week 2, week 2 and 4, and baseline and week 6 were statistically significant, they were comparable across both the groups. The change in score from week 4–6 in the moclobemide group was statistically less significant than that in the imipramine group (p<0.05 vs p<0.001) (Table 3).

**Table 3:** Mean changes and percentage reduction in the scores on the Montgomery–Asberg Depression Scale (MADRS) in ‘treatment completers’

	Moclobemide (*n*=21)		Imipramine (*n*=19)	
		
Duration (in weeks)	Mean changes	Reduction (%)	Mean changes	Reduction (%)
Baseline–week 2	7.57	20.75*	10.42	28.73*
Week 2–week 4	6.43	22.24*	7.21	27.90*
Week 4–week 6	4.05	18.02**	6.47	34.78*
Baseline–week 6	18.05	49.49*	24.11	66.49*

* p<0.001;

** p<0.05

As shown in Table 4, the mean total score on MADRS in the ‘intent to treat’ group fell from 36.83 (SD±6.31) at baseline to 19.96 (SD±12.23) at week 6 in the moclobemide group, and from 37.00 (SD±5.66) to 16.08 (SD±10.61) in the imipramine group. Although the changes between baseline and week 2, week 2 and 4 and baseline and week 6 were statistically significant, they were comparable across the two groups. The change of score in week 4–6 in the moclobemide group was statistically less significant than that in the imipramine group (p<0.05 vs p<0.001).

**Table 4:** Mean changes and percentage reduction in the scores on the Montgomery–Asberg Depression Scale (MADRS) in the ‘Intent to treat patients’ group

	Moclobemide (*n*=24)		Imipramine (*n*=24)	
		
Duration (in weeks)	Mean changes	Reduction (%)	Meanchanges	Reduction (%)
Baseline–week 2	7.00	19.00*	11.92	32.21*
Week 2–week 4	6.04	20.25*	5.16	20.57*
Week 4–week 6	3.83	16.10**	3.84	19.28*
Baseline–week 6	16.87	45.81*	20.92	56.53*

* p<0.001;

** p<0.05

### Responders

Sixty-two per cent of patients belonging to the moclobemide group and 84% in the imipramine group were responders on the HDRS. There were 52.38% responders in the moclobe-mide group and 73.68% in the imipramine group on MADRS. Overall, the patients on imipramine responded better than those on moclobemide at the end of 6 weeks.

### Safety and tolerability

Moclobemide appears to be better tolerated than imipramine. Anticholinergic side-effects were reported less often by patients on moclobemide as compared with imipramine—dry mouth in 6 patients (20%) vs 9 (30%), constipation in 1 (3.33%) vs 11 (36%), blurred vision in 3 (10%) vs 6 (20%) (Table 5).

**Table 5:** Treatment-emergent side-effects

Side-effect	Moclobemide (*n*=30)	Imipramine (*n*=30)
Insomnia	1 (3.33)	1 (3.33)
Dry mouth	6 (20.00)	9 (33.33)
Blurred vision	3 (10.00)	6 (20.00)
Headache	1 (3.33)	3 (10.00)
Dizziness	2 (6.66)	6 (20.00)
Sweating	0 (0)	4 (13.33)
Nausea	2 (6.66)	1 (3.33)
Constipation	1 (3.33)	11 (36.66)
Postural hypotension	0 (0)	2 (6.66)
Mild sedation	1 (3.33)	0 (0)
Difficulty in swallowing	0 (0)	2 (6.66)

Values in parentheses are percentages.

## DISCUSSION

This study compares the efficacy and safety of moclobemide with that of imipramine in the treatment of major depressive disorder in Indian patients. The drugs were found to have comparable efficacy as measured by the HDRS and MADRS. Studies comparing the efficacy of moclobemide with that of other TCAs such as imipramine^2^,^3^,^11^ and amitriptyline^27^ have reported similar results. The rapidity of improvement as measured by the decrease in scores on the HDRS and MADRS in successive weeks were similar in both the groups. These results are in accordance with that of previous studies.^2^,^3^,^11^ The change in scores in week 4–6 was found to be statistically less significant in the moclobemide than the imipramine group (p=0.05 vs p=0.001). No previous study has found such a change. It could be because common side-effects encountered with imipramine therapy decrease considerably by the end of this period. This may be responsible for the greater decrease in the scores. Studies with double-blind measures and a larger sample size can solve this riddle. Rimon *et al*.^2^ reported faster onset of antidepressant action in the moclobemide than the imipramine group as measured by assessment of investigators on a scale of ‘Very Good’ to ‘No Change’. However, the onset of action was similar in both the groups as measured by a decrease in the HDRS scores. The present study also found a similar onset of action in both the groups as measured by a reduction in the HDRS and MADRS scale scores. It strengthens the observation that measurements of onset of action should be done in terms of scale measurements rather than simple clinical impressions.

Although the change in scores from the baseline to week 6 was comparable on both the HDRS and MADRS in both the groups, there were a significantly larger number of responders in the imipramine group. This could be due to the greater reduction in the HDRS and MADRS scores in weeks 4–6. These results might suggest somewhat better response for imipramine in achieving clinical response. However, these findings should be interpreted in the light of the fact that definition of clinical response in this study was an arbitrary one and this was an open, non-blind, randomized trial.

As observed in other studies,^2^,^3^,^11^ anticholinergic side-effects such as dry mouth, constipation, blurring of vision were more common in patients treated with imipramine than with moclobemide. The most common side-effects encountered with moclobemide were dry mouth (20%) and blurred vision (10%). However, no patient discontinued treatment due to these adverse effects. On the other hand, 6 patients in the imipramine group discontinued treatment due to the adverse effects. Hence, the overall tolerability of moclobemide is much better than that of imipramine as shown in earlier studies.^2^,^3^ Highly selective affinity towards MAO-A and almost no affinity for muscarinic, dopa-minergic, serotoninergic, opioid or benzodiazepine receptors might be responsible for its greater tolerability and fewer side-effects.

To conclude, moclobemide is a safe and effective antidepressant with a favourable side-effect profile.
